# Improved Mechanical Properties of Graphene/Carbon Fiber Composites via Silanization

**DOI:** 10.1021/acsaenm.4c00236

**Published:** 2024-07-08

**Authors:** Xudan Yao, Jason H. Hui, Ian A. Kinloch, Mark A. Bissett

**Affiliations:** Department of Materials, Henry Royce Institute, National Graphene Institute, University of Manchester, Oxford Road, Manchester M13 9PL, U.K.

**Keywords:** graphene, electrochemical exfoliation, silanization, CFRP composites, mechanical properties

## Abstract

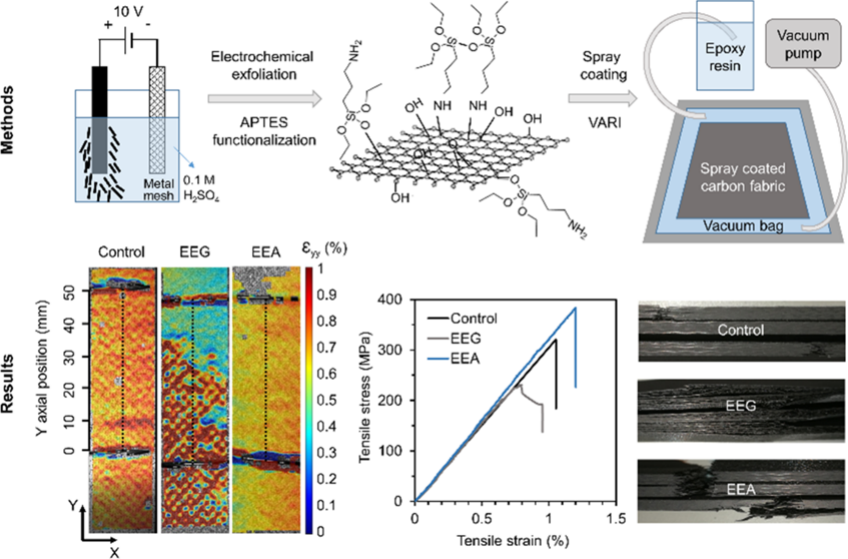

Despite their excellent mechanical performance, carbon fiber-reinforced polymer (CFRP) composites are limited by the interfacial properties due to the inherent nature of laminated structures. One way to modify the interface is by the inclusion of nanomaterials. Here, we use electrochemical exfoliation to produce graphene (EEG) flakes that have hydroxyl and epoxy functional groups. To further improve the interfacial bonding, silanization was carried out on graphene with 3-aminopropyl triethoxysilane, and then, EEA flakes were achieved. Both flakes were dispersed in ethanol and spray-coated onto carbon fibers, followed by vacuum-assisted resin infusion to make hybrid composites. Testing of their mechanical properties showed that EEG flakes tend to act as points of stress concentration, which accelerated the delamination, while the EEA flakes improved interfacial properties owing to the covalent bonding. As a result, with only 0.5 wt % EEA flakes spray-coated onto the carbon fibers, the tensile and flexural strength of graphene/carbon fiber composites improved by 17.6 and 5.4%, respectively. The combination of electrochemical exfoliation, silanization, spray coating, and vacuum-assisted resin infusion enables large-scale hybrid composite fabrication without size or shape limitations, without weakening the CFs or carbon fabric patterns, and is suitable for continuous production. This process has proven to be practical and attractive for engineering applications.

## 1 Introduction

In recent decades, the remarkable specific strength and stiffness of the carbon fiber-reinforced polymer (CFRP) composites have drawn much attention and made them widely applicable in diverse fields, such as aerospace, automotive, and civil engineering. Despite their excellent mechanical behavior, the interfacial properties are a key parameter which limits their performance. One approach to address this is with the development of new composite fabrication methods, e.g., 3D weaving, stitching, or z-pinning to prevent the delamination and improve the impact resistance while facing challenges such as penetration, defects, porosity, etc.^1−3^ On the other hand, other methods without introducing the z-direction fibers have been proposed to improve the interfacial properties, such as the addition of nanomaterials (e.g., graphene-related materials,^4−16^ carbon nanotubes (CNTs),^14,16−19^ cellulose,^20^ MXene,^21^ etc.) and the chemical modification of carbon fibers (CFs).^22,23^

In particular, graphene-related materials (GRMs), owing to their exceptional mechanical, electrical, and thermal properties, have been widely investigated.^15,16^ Some researchers decorated CFs with GRMs to improve the interfacial properties, among which the electrophoretic deposition (EPD) and fiber sizing methods were used widely. Gangineni et al.^8^ studied several GRMs and found that graphene carboxyl (G-COOH) performs the best, which contributed to 9.6 and 22.9% improvements in flexural strength and interlaminar shear strength (ILSS), respectively. Similarly, Bhanuprakash et al.^11^ reported 25 and 47% improvements in flexural strength and ILSS with CFs coated by graphene oxide (GO) via EPD. Regarding the fiber sizing method, GRMs were dispersed in the fiber sizing and then coated onto the CFs, which also contributed to significant enhancements in flexural properties and ILSS.^6,7,10^ In addition, Zhang et al.^14^ reported the synergistic effect of using CNTs, GO, and Ag nanoparticles, combining electrodeposition and chemical grafting, and the CF-Ag-GO–CNT composite resulted in a 69.7% higher interfacial shear strength (IFSS) compared with the untreated CFRP composites.

Apart from decorating CFs, GRMs have also been utilized in modifying the epoxy resin. Adak et al.^5^ functionalized GO with polyethylenimine and then dispersed it in epoxy; ∼60 and ∼67% improvements on tensile and flexural strength of the composites were achieved. Kim et al.^12^ functionalized graphene nanoplatelets (GNPs) noncovalently via π–π interactions with poly(4-aminostyrene) (PAS), and as a result of better dispersion and crack bridging, 252% and 142% improvements in ILSS and fracture toughness were obtained with 4 wt % PAS-GNPs. Du et al.^13^ made partially cured 1 wt % graphene/epoxy interleaves and then co-cured them with CFRP composites, which achieved a remarkable 140% increase in mode I interlaminar fracture energy. Qu et al.^24^ introduced different loadings of GO to modify the epoxy, and the ILSS reached the peak value at the 0.2 wt % loading, where ∼18% improvement was achieved. Hung et al.^9^ introduced GO into CFRP composites by two methods, dispersing into the epoxy resin and coating onto the surface of the carbon-woven fabric via EPD, and the latter one achieved a more significant improvement in the mechanical properties of composites.

As literature has illustrated,^5,10,25−27^ chemical functionalization is an effective route to strengthen the interface via covalent bonding, among which silanization can introduce functional moieties like ethylene, amine, epoxy, thiohydroxy, etc., using silane-coupling agents via amidation or esterification reactions, which have been applied in both nanocomposites and hybrid composites.^25−30^ Khan et al.^27^ functionalized GNPs through oxidation followed by silanization and then dispersed the fillers into the matrix to reinforce CFRP composites, resulting in improved ILSS and tensile and flexural strengths. We have previously applied two types of silane-coupling agents to GO for amino and epoxy functionalization, where the former one contributed more to the strength and stiffness, indicating a better interfacial stress transfer in the GO/epoxy nanocomposites.^26^

**Figure 1:**
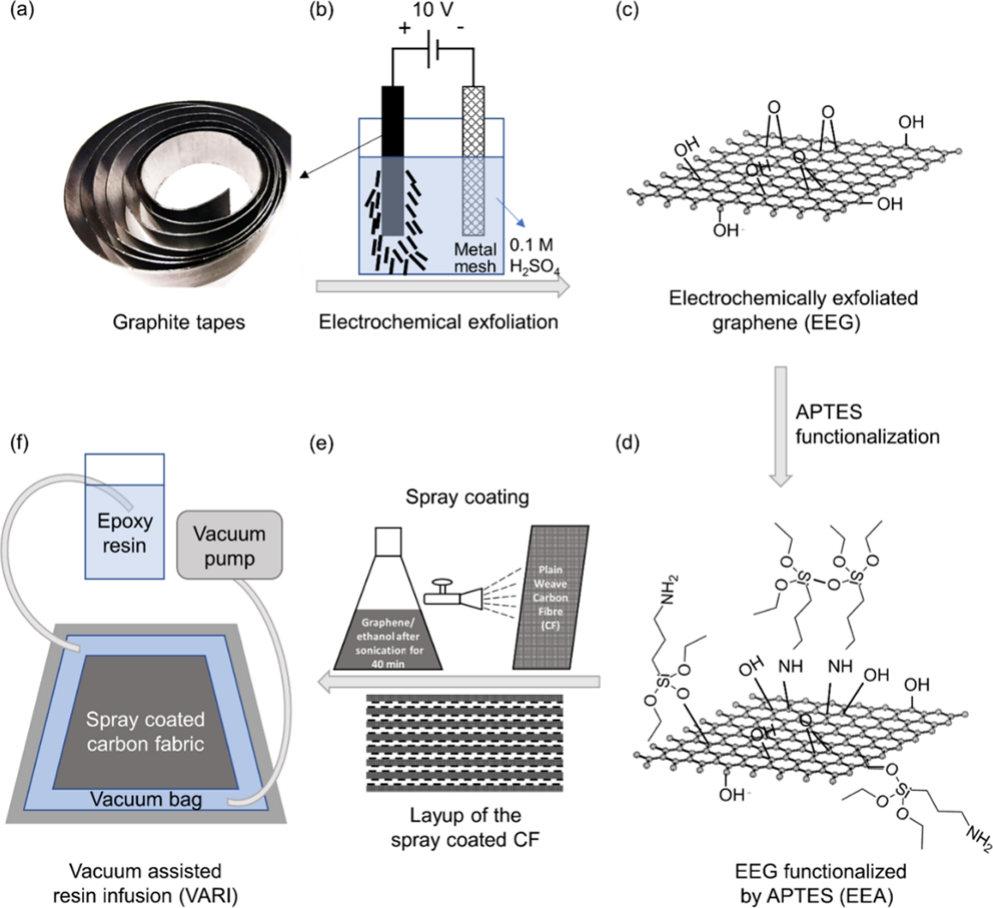
(a) Photograph of the graphite tape. (b) Schematic of the anodic electrochemical exfoliation. (c) Structure of electrochemically exfoliated graphene (EEG). (d) EEG functionalized by APTES (EEA). Schematic of the (e) spray coating process and (f) vacuum-assisted resin infusion (VARI).

Electrochemical exfoliation of graphite is considered one promising route for the mass production of relatively large flakes, and it has been of interest in recent years.^31−36^ The procedure involves graphite working as the anode for intercalation and a metal mesh as the cathode, an electrolyte to assist the exfoliation, and a power supply to provide the current.^37,38^ With a constant voltage applied, the positively charged graphite layers attract the negatively charged anions, with the intercalation rapidly occurring and hydroxyl groups also forming, and possibly a secondary chemical reaction leading to epoxides C–O–C,^38^ as shown in Figure 1. Regarding the electrolyte, sulfuric acid stands out with its high exfoliation efficiency.^33,39^ However, mass-produced graphene flakes with chemical functionalization have not yet been applied to CFRP composites with a continuous production capability for engineering applications.

In this work, electrochemically exfoliated graphene (EEG) flakes and amine-functionalized EEG flakes are prepared and systematically characterized before being utilized in CFRP composites. Regarding the composite fabrication, our previous work^40,41^ proposed a method combining spray coating with vacuum-assisted resin infusion to achieve CFRP composites with preferred in-plane aligned graphene flakes, which possess simplicity and flexibility with potential for industrialization, which will be continuously used in this work. Also, the effect of the filler loading was investigated,^40^ and CFs spray-coated with 0.5 wt % (relative to the CFs) graphene showed the best mechanical performance, and they are selected in this study.

## 2 Experimental Section

### 2.1 Materials

Graphite tapes (100 mm × 15 mm × 0.5 mm) were obtained from Gee Graphite Ltd., which were used for synthesizing the graphene flakes. Plain weave carbon fibers were purchased from Sigmatex, which used the Hexcel HexTow carbon fiber, with the density of 199 g/m^2^. Low-viscosity Araldite LY564 and Aradur 2954 were received from Huntsman. 3-Aminopropyl triethoxysilane (APTES), sulfuric acid (H_2_SO_4_), and ethanol were purchased from Fisher Scientific and used as received.

### 2.2 Electrochemical Exfoliation of Graphene Flakes

The graphene flakes were first prepared by electrochemical exfoliation. Briefly, 0.1 M H_2_SO_4_ solution was used as the electrolyte, with the graphite tape (anode, Figure 1a) and a steel mesh (cathode) placed vertically in the reaction cell, as shown in Figure 1b. A constant voltage (10 V) was applied to the electrodes for 10 min. After the exfoliation, the EEG flakes (Figure 1c) were filtered and washed by deionized (DI) water until neutral (pH 7) followed by vacuum drying (50 °C) overnight.

### 2.3 Silanization of EEG Flakes

The EEG flakes were functionalized by an aminosilane, APTES, using the method developed previously.^26^ Briefly, 6 g of APTES and 0.6 g of EEG flakes were added into a 500 mL mixture of DI water and ethanol (1:3 by volume), followed by 1 h of ultrasonication. Afterward, the whole mixture was refluxed in a water bath at 70 °C for 4 h and then filtered and washed with the same mixture of DI water and ethanol until the pH stabilized at 7. After being dried overnight in the vacuum oven at 50 °C, the EEG flakes functionalized with APTES were achieved and named as EEA flakes (Figure 1d).

### 2.4 Fabrication of Composites

Spray coating, followed by a resin infusion method, was selected for the composite fabrication. After sonicating the mixture of graphene flakes and ethanol (∼5 mg/mL) for 40 min, it was sprayed onto the plain weave carbon fibers (Figure 1e), which was detailed in our previous work.^41^ Then, the carbon fibers were left overnight to let ethanol evaporate completely, followed by vacuum-assisted resin infusion (VARI, Figure 1f) to fabricate the composites. Composite laminates (300 mm × 200 mm), made of 8 plies of the carbon fabric with a quasi-isotropic layup [(0/90)/(±45)]_2s_, were cured at 80 °C for 2 h, followed with curing for 140 °C for 8 h. CFRP composites spray-coated with pure ethanol and EEG and EEA flakes at the loading of 0.5 wt % CF were prepared and denoted as the control and EEG and EEA composites.

### 2.5 Characterization

Scanning electron microscopy (SEM) accompanied by energy-dispersive X-ray spectroscopy (EDS) was used to characterize the morphology and elemental distribution of CFs and graphene flakes, using a TESCAN MIRA3 SC. A JPK NanoWizard atomic force microscope (AFM) was adopted for thickness characterization of the graphene flakes using the QI mode. A PANalytical X’Pert Pro diffractometer equipped with a Cu Kα radiation source was used to obtain the X-ray diffraction (XRD) patterns in the range of 2θ = 3.02 to 99.98°. Raman spectroscopy was performed using a Renishaw InVia Raman system (λ = 633 nm). Fourier transform infrared (FTIR, Thermo Electron Corporation, Nicolet 5700) spectroscopy was used to identify the functional groups on the EEG and EEA flakes. Transmission electron microscopy (TEM, FEI Tecnai G2 20, LaB_6_) was used to observe bright-field images and diffraction patterns of the graphene flakes. X-ray photoelectron spectroscopy (XPS) was performed using an ESCA2SR spectrometer (ScientaOmicron GmbH) via monochromated Al Kα radiation (1486.6 eV, 20 mA emission at 300 W, 1 mm spot size) with a base vacuum pressure of ∼1 × 10^–9^ mbar. Charge neutralization was achieved using a low-energy electron flood source (FS40A, PreVac). Binding energy scale calibration was performed using C–C in the C 1s photoelectron peak at 285 eV. Analysis and curve fitting were performed using Voigt approximation peaks using CasaXPS.^42^

### 2.6 Mechanical Testing

The tensile properties of hybrid composites were evaluated based on ASTM D3039, with a specimen size of 250 mm × 25 mm × 2 mm and a cross-head speed of 2 mm/min. The test was undertaken in the environmental lab with a constant temperature and relative humidity of 23 °C and 50%. A digital image correlation (DIC) system accompanied by a video extensometer with the gauge length calibrated at 50 mm was employed to monitor the strain distribution and extension during the test.

Four-point bending tests were performed for flexural properties according to ASTM D7264, with the specimen size of 100 mm × 12.7 mm × 2 mm and the support span (*L*) and load span set at 67.2 and 33.6 mm, respectively. The testing rate (*R*) of 3.59 mm/min was calculated based on the equation (*R* = 0.167*ZL*^2^/*d*) from ASTM D6272, where *d* is the depth (thickness) of the beam (mm) and *Z* is the straining rate of the outer fibers (0.01 mm/mm min).

## 3 Results and Discussion

### 3.1 Characterization of EEG and EEA Flakes

#### 3.1.1 Aspect Ratio of the Flakes

**Figure 2:**
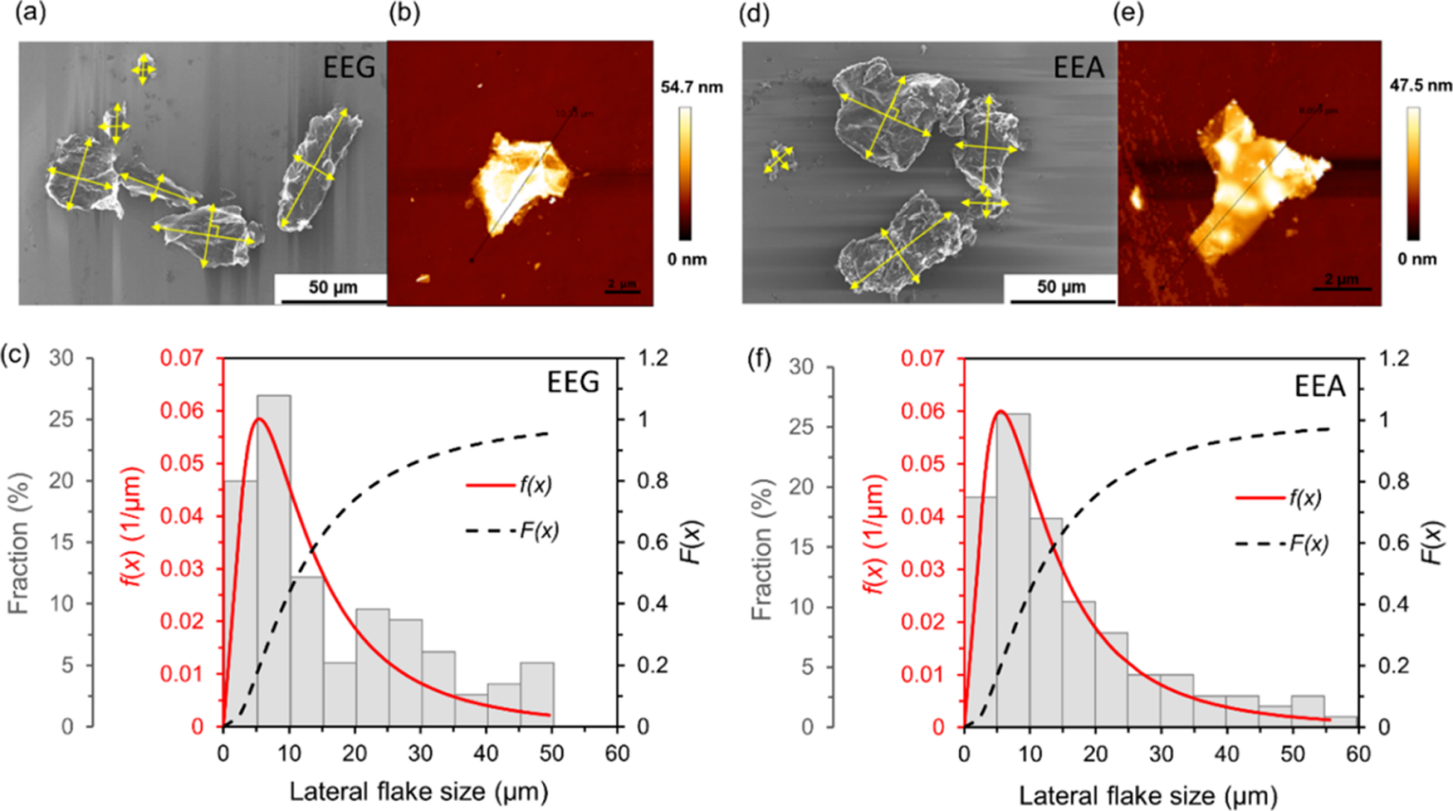
SEM and AFM images, as well as histograms and lateral flake size distribution functions, of (a–c) EEG and (d–f) EEA flakes.

The aspect ratio of the filler is a vital parameter that dominates the efficiency of stress transfer, i.e., reinforcement performance.^16,43,44^ Young et al.^44,45^ suggested that strong graphene–polymer interfaces and aligned GNPs with high aspect ratios contribute to the best reinforcement in nanocomposites. In order to clarify the role of flake dimension on the mechanical performance of the composites, lateral size distributions of both EEG and EEA flakes were first evaluated. SEM images of more than 100 EEG and 100 EEA flakes have been taken, with both the length and width (perpendicular to the length measurement) measured, as shown in Figure 2a,d. Then, the average value was set as the lateral size. Histograms of the EEG and EEA flakes distributions are summarized in Figure 2c,f, along with the probability density functions (*f*(*x*)) and cumulative distribution functions (*F*(*x*)) obtained based on the log-normal distribution equations
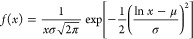
2
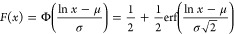
3where *x* represents the lateral flake size, and μ and σ represent the mean and standard deviations of ln(*x*), respectively. Φ is the cumulative distribution function of the standard normal distribution, and erf is the complementary error function.

The lateral sizes (*l*) of both EEG and EEA flakes distribute within a relatively wide range, from several micrometers to 50–60 μm, with the average sizes of 16.3 ± 13.3 and 15.7 ± 13.1 μm, respectively. Similarly, the thickness (*t*) distribution of the flakes was characterized by AFM, as shown in Figure 2b,e, and the average values are 114.3 ± 145.3 and 110.5 ± 131.2 nm for EEG and EEA flakes, respectively, with the detailed distribution shown in the Supporting Information (SI, Figure S1). As a consequence, the average aspect ratios, *s* = *l*/*t*, sit at 142.6 and 142.1 for EEG and EEA flakes, respectively. It indicates that the lateral size and thickness of both flakes varied in a relatively wide range, and the functionalization procedure did not alter the size significantly.

#### 3.1.2 Morphology and Chemical Composition of the Flakes

**Figure 3:**
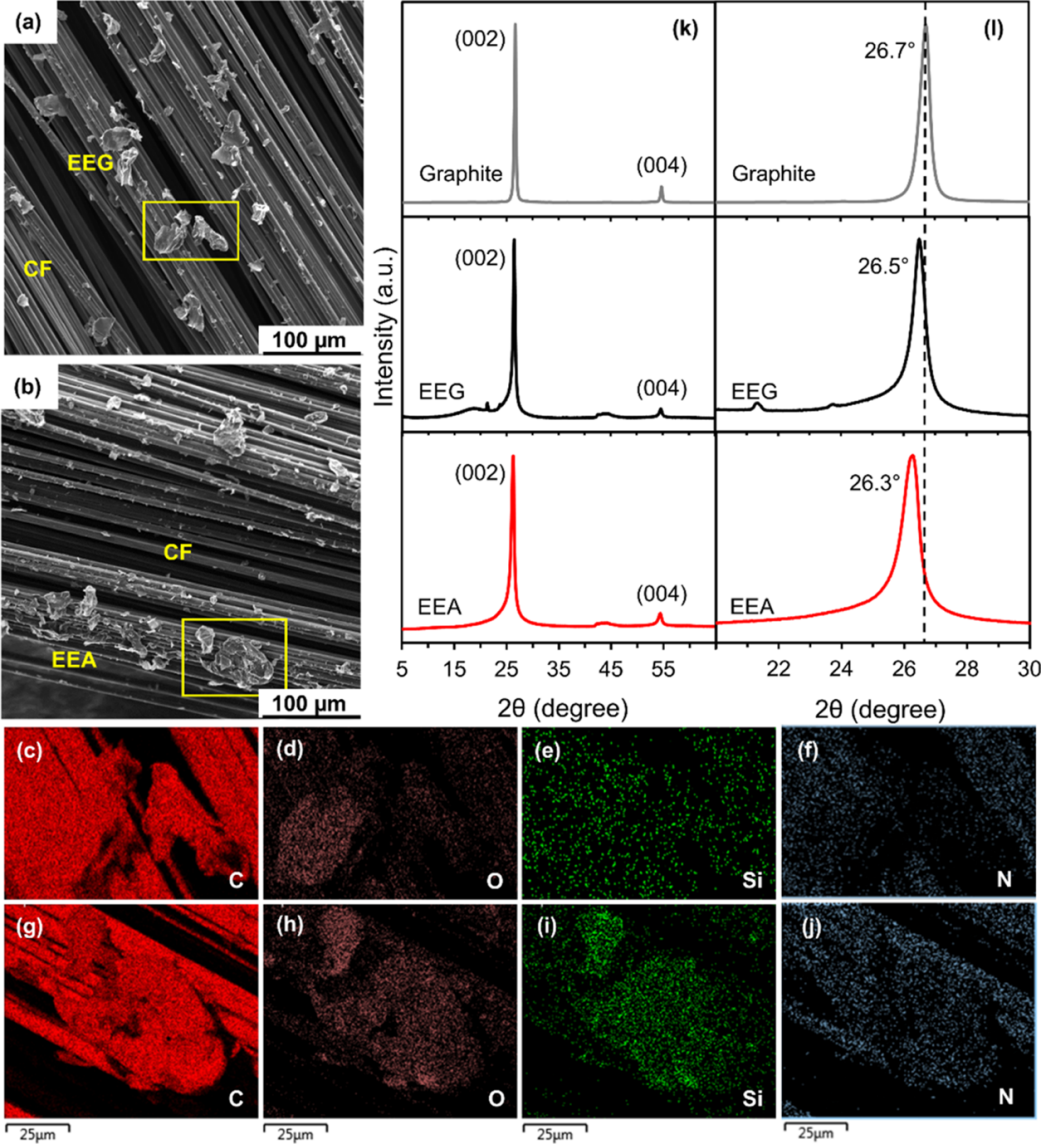
SEM images and EDS elemental mapping of carbon (C, red), oxygen (O, pink), silicon (Si, green), and nitrogen (N, blue) for carbon fibers spray-coated by (a, c–f) EEG and (b, g–j) EEA flakes. XRD characterization of the original graphite tape, EEG, and EEA: (k) whole spectra and (l) the high-resolution (002) peak.

In order to verify the chemical functionalization and understand the chemical composition, carbon fibers spray-coated by EEG and EEA flakes were characterized by SEM and EDS analyses (Figure 3a–j). The result suggests that weight fractions of oxygen (O), silicon (Si), and nitrogen (N) elements increased after the APTES functionalization (with the detailed information summarized in Table S1). The XRD patterns (Figure 3k,l) show that the graphitic peak (002) shifts to lower angles (2θ) after both electrochemical exfoliation and silanization, with the value decreased from 26.7° (original graphite tape) to 26.5° (EEG flakes) and finally to 26.3° (EEA flakes). This indicates the expansion of graphite layers^32^ caused by the introduction of oxygen and silane groups (as seen in Figure 1c,d), thus confirming the success of the exfoliation and functionalization. Also, in the Raman spectra (Figure S2), the increased *I*_D_/*I*_G_ and fwhm of the 2D peak indicate the increased level of disorder introduced by the functionalization,^46^ with details given in the Supporting Information.

**Figure 4:**
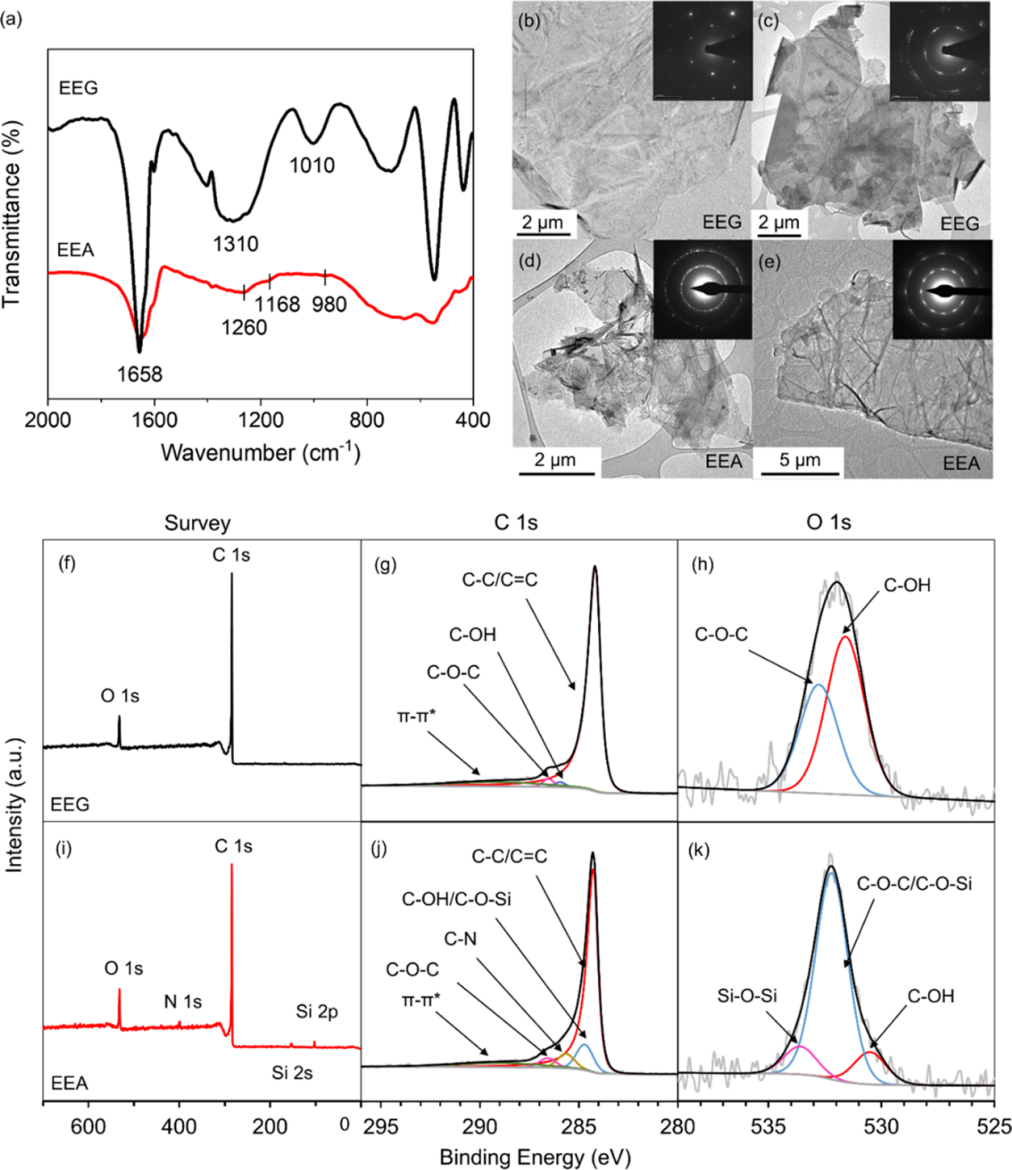
(a) FTIR spectra of the EEG and EEA flakes. Bright-field TEM images of (b, c) EEG and (d, e) EEA flakes, embedded with their corresponding selected area electron diffraction (SAED) patterns. Survey and high-resolution C 1s and O 1s spectra of (a–c) EEG and (d–f) EEA flakes obtained from XPS.

TEM bright-field images and the corresponding selected area electron diffraction (SAED) patterns (Figure 4b–e) indicate that both EEG and EEA flakes vary from few-layer to many-layer graphene sheets with a wrinkled morphology. The SAED pattern of the thin flakes demonstrated a clear 6-fold symmetry owing to the hexagonal structure of carbon atoms, while for the thick flakes, ringlike structures appeared due to the higher stacking disorder.^47^

FTIR spectroscopy and XPS were employed to analyze the chemical composition and bonding types of EEG and EEA flakes. In FTIR spectra (Figure 4a), characteristic C=C stretching (∼1658 cm^–1^) bands are present in both curves, attributed to the existence of the aromatic ring,^48,49^ while there is no obvious band in the 1700–1740 cm^–1^ range, indicating that there are a few C=O-related carbonyl and carboxyl groups, as we previously reported,^38^ for either EEG or EEA flakes. The EEG curve shows the characteristic hydroxyl band (C–OH, 1310 cm^–1^) and the epoxy vibrational band (C–O–C, 1010 cm^–1^),^50−52^ which were largely weakened in the EEA curve. In contrast, C–N (1260 cm^–1^), Si–O–C (1168 cm^–1^), and Si–O–Si (980 cm^–1^) vibrational bands^53−60^ are observed, as a result of chemical reactions between APTES (amino or silyl groups) and EEG flakes (epoxy or hydroxyl groups).^55^

Furthermore, XPS results shown in Figure 4f,i indicate that N 1s (399 eV), Si 2s (153.5 eV), and Si 2p (102.5 eV) peaks appeared in EEA, accompanied by the C/O ratio decreased from 11.8 to 10.0 (Table S2), indicating the reactions between oxygen groups of EEG and amine groups of APTES during the functionalization.^61^ High-resolution C 1s spectra (Figure 4g,j) show the representative graphite carbon (C–C/C=C), hydroxyl (C–OH), and epoxy (C–O–C) groups,^26^ as well as the π–π* bonds corresponding to the conjugated structure.^38,62^ The appearance of C–O–Si, Si–O–Si, and C–N groups (Figure 4j,k), as well as the content of C–OH decreasing from 67.1 to 13.2% (Table S3), further illustrates the chemical reactions and is consistent with the FTIR results. Detailed elemental and bonding comparisons of EEG and EEA flakes can be found in the SI.

### 3.2 Mechanical Properties

#### 3.2.1 Tensile Properties

**Figure 5:**
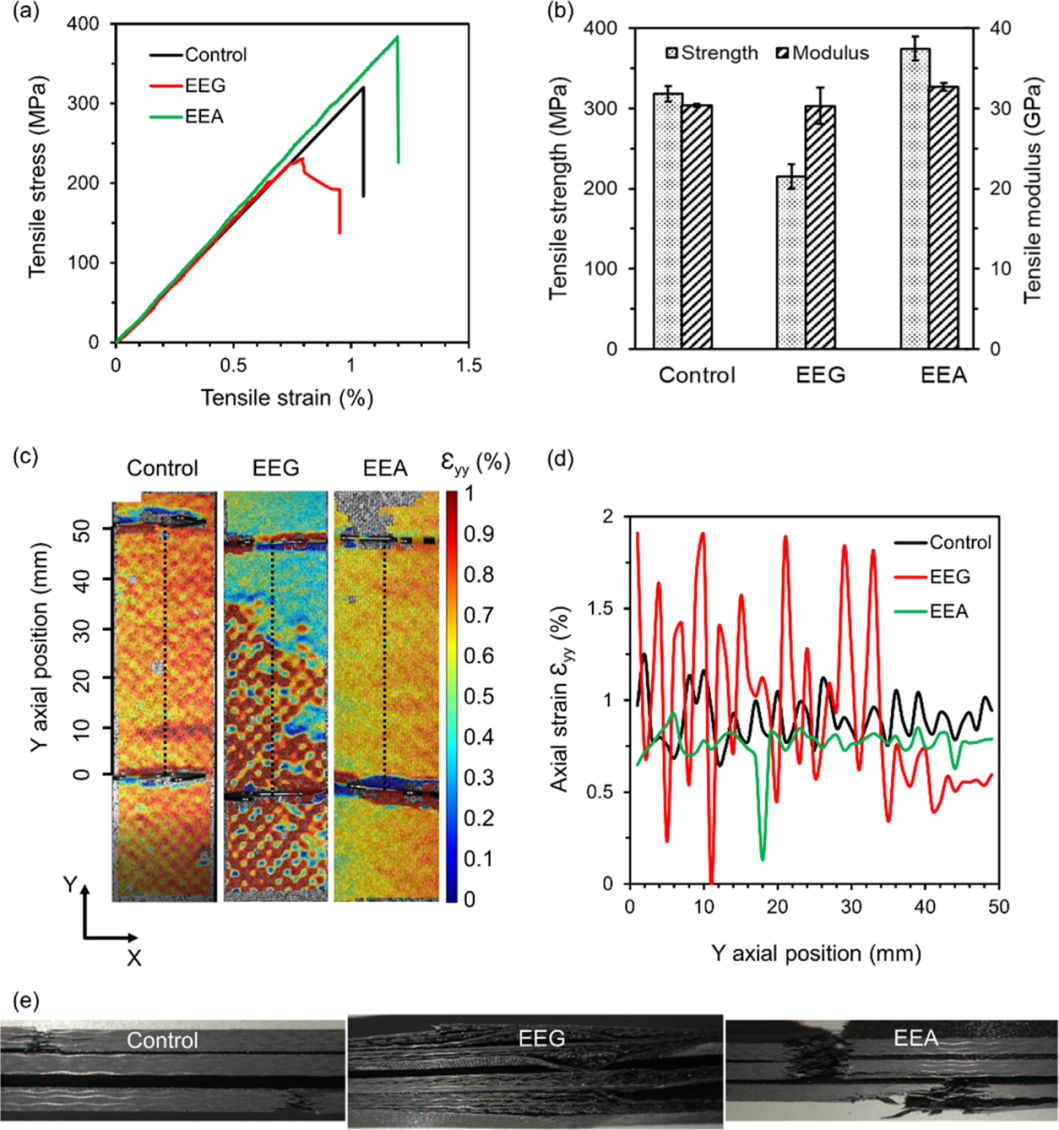
(a) Tensile stress–strain curves of the control carbon fiber-reinforced polymer (CFRP) composites, as well as composites with carbon fibers spray-coated by 0.5 wt % EEG and EEA flakes. (b) Tensile strength and modulus of the composites. (c) Axial strain maps of the control and EEG and EEA composites under 90% ultimate tensile stress, recorded by the digital image correlation (DIC) system. (d) The corresponding axial strain ε_*yy*_ distribution along the *Y* direction (marked by dotted lines in panel (c)) between the extensometer. (e) Images of failed composites across the out-of-plane direction, showing severe delamination in EEG composites, which was significantly reduced for EEA composites.

The representative stress–strain curves of the control and EEG and EEA composites under tension are shown in Figure 5a. Both the control and EEA-reinforced CFRP composites behaved linearly throughout the whole tensile procedure, while EEG exhibited a nonlinear behavior starting from ∼0.65%. This can be attributed to the weak interfacial properties between the EEG flakes and matrix, and delamination occurred during the tensile procedure, leading to a 32.4% loss of tensile strength (Figure 5b). The tensile modulus, which represents the elastic properties of the composites, remained unchanged with the addition of EEG flakes, as shown in Figure 5b. By comparison, EEA samples possess amine groups, which are capable of reacting with the epoxy resin and forming covalent bonding, similar to the reaction between the epoxy and the hardener.^26^ Consequently, the interfacial properties and load transfer efficiency were largely improved; as a result, the tensile strength increased from 318.3 ± 9.4 to 374.4 ± 14.7 MPa, and the modulus increased from 30.3 ± 0.2 to 32.7 ± 0.5 MPa, indicating an improvement of 17.6 and 7.9%, respectively, compared to the control composites.

In order to further understand the failure mechanism, a digital image correlation (DIC) system was applied to monitor the strain distribution on the composites during the test. Both the control and EEA-reinforced CFRP composites experienced a uniform axial strain distribution (Figure 5c,d) during the tensile procedure and fractured with sharp surfaces and fiber breakage, with no obvious delamination (Figure 5e), while EEG showed a large variation of strain distribution over the sample (Figure 5c,d) and failed with severe delamination, which can be seen across the out-of-plane direction (Figure 5e). The uniform strain distribution represents a uniform load transfer across the sample, indicating excellent interfacial properties. For the EEA samples, the interfacial properties are attributed to the strong connections between the EEA flakes and the matrix. On the contrary, without chemical bonding forming, EEG flakes act as points of stress concentration,^63^ leading to the severe delamination.

#### 3.2.2 Flexural Properties

**Figure 6:**
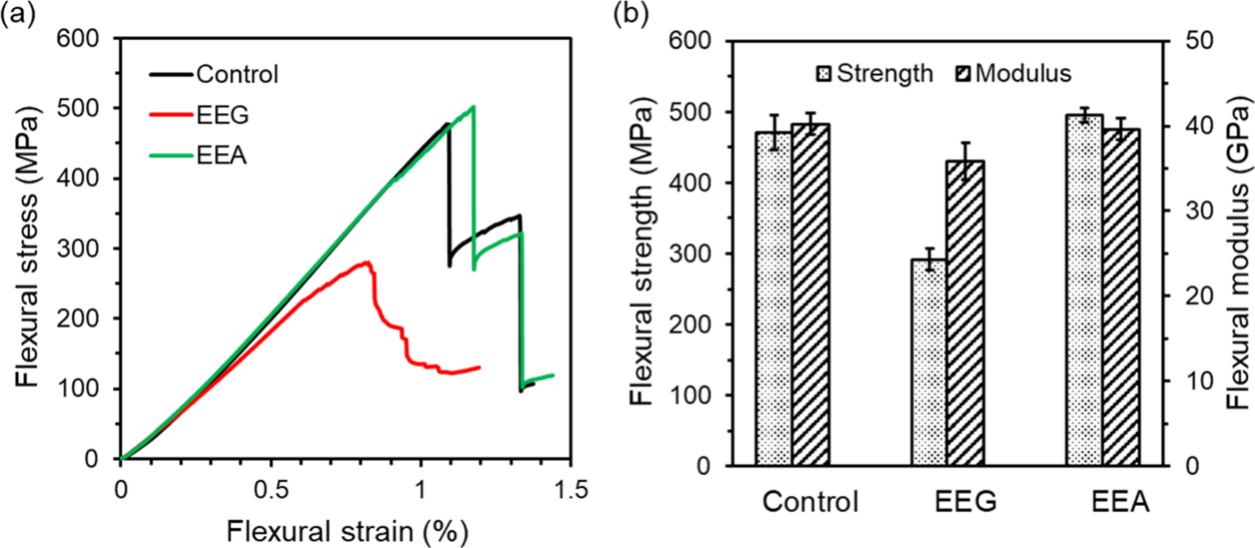
(a) Flexural stress–strain curves and (b) flexural strength and modulus of the control and EEG and EEA composites.

Figure 6a shows representative flexural stress–strain curves for the control and EEG and EEA composites. Similar to the tensile performance, stresses of both the control and EEA composites increased linearly with the strains until ultimate flexural stresses were achieved, while the stress of EEG composites increased nonlinearly with the strain starting from ∼0.6% due to the occurrence of the delamination. As previously discussed in the tensile properties, EEG flakes led to severe stress concentrations; as a result, the flexural strength and modulus decreased by 38.0 and 8.8%, as shown in Figure 6b. After the amine grafting, the covalent bonding between EEA flakes and the matrix contributed to strong interfacial connections, which improved the flexural strength by 5.4%, compared to the control composites (Figure 6b).

#### 3.2.3 Comparison with Literature

From a comparison of the CFRP composites reinforced by GRMs (Table 1), in general, GRMs contribute to improve the mechanical properties of the composites either by dispersing in the epoxy or coating/grafting onto carbon fibers, particularly with functionalization. When it comes to the chemical treatment on carbon fibers, acid treatment and electrophoretic deposition (EPD) led to 15.9 and 13.6% reductions in tensile strength^14^ due to the triggered defects. In this work, graphene flakes were successfully exfoliated by electrochemical exfoliation and then functionalized via silanization, followed by spray coating onto carbon fibers and resin infusion to fabricate the composites. The whole procedure can be scaled up for a large amount of graphene production and large structure fabrication, without weakening carbon fibers or damaging the carbon fabric patterns and with no size/shape limitations, which is promising for engineering applications. In addition, more uniformly distributed particle sizes would potentially improve the distribution during spray coating, which could translate into a significant increase in the mechanical properties of the composites in future applications.

**Table 1:** Comparison of CFRP Composites Reinforced by GRMs^a^

material	comment	functionalization	loading (wt %)	strength	improvement (%)	ref
graphene	EPD^b^	no		flexural, ILSS^c^	4.6, −3.5	(8)
		hydroxyl			5.1, 13.8	
		carboxyl			9.6, 22.9	
	graphene/epoxy interleaves	no	1	G_IC_^d^	140	(13)
	spray coating	silanization	0.5	tensile, flexural	17.6, 5.4	this work
GO^e^	graft onto the acyl chloride-functionalized CF^f^	amino		IFSS^g^	36.4	(4)
	disperse in the epoxy	polyethylenimine	0.3	tensile, flexural	58–62, 65–70	(5)
		no	0.2	ILSS	17	(24)
	disperse in fiber sizing	no	5	ILSS, tensile	12.7, 34.2	(6)
		triazine derivatives	1	ILSS, flexural	100.2, 78.3	(10)
	EPD	no		flexural	37.3	(9)
	disperse in the epoxy	no			18.0	
	EPD	no		ILSS, flexural	47, 25	(11)
	EPD + chemical grafting	Ag nanoparticles + carbon nanotubes		IFSS, CF tensile	69.7, −1.9	(14)
GNP^h^	immerse coating	no		90°/0° flexural, ILSS	52/7, 19	(7)
	disperse in the epoxy	poly(4-aminostyrene)	4	ILSS, G_IIC_^i^	252, 142	(12)
		amine	0.5	tensile, ILSS	97, 27.4	(27)

a Graphene-related materials.

b Electrophoretic deposition.

c Interlaminar shear strength.

d Fracture toughness mode I.

e Graphene oxide.

f Carbon fiber.

g Interfacial shear strength.

h Graphene nanoplatelet.

i Fracture toughness mode II.

## 4 Conclusions

This work demonstrated the potential of combining electrochemical exfoliation and silanization for the large-scale production of functionalized graphene flakes, which could be used to strengthen CFRP composite structures via spray coating and vacuum-assisted resin infusion, without weakening carbon fibers or damaging the carbon fabric patterns. Combined characterization not only qualified but also quantified the formed functional groups. The lateral size and thickness of both EEG and EEA flakes were analyzed via SEM and AFM, which showed comparable values; thus, the effect of the aspect ratio could be eliminated during the comparison. As a consequence, the mechanical performance difference between the EEG and EEA composites is dominated by silanization. As a result of improved interfacial properties, the tensile and flexural strengths improved by 17.6 and 5.4%, respectively, compared with the control composites, with only 0.5 wt % EEA flakes spray-coated onto the carbon fibers. The whole procedure is shown to be practical and attractive for engineering applications. In the future, the effect of the aspect ratio and particle size could be investigated, and specific particle sizes could be selected for further improvement.

## Supporting Information Available

Thickness distribution, EDS elemental analysis, Raman spectra, and XPS analysis of EEG and EEA flakes (PDF)
