# Supplementary material for: Improved Mechanical Properties of Graphene/Carbon Fiber Composites via Silanization
Source: ACS Appl Eng Mater. 2024 Jul 8;2(7):1836–44. doi: 10.1021/acsaenm.4c00236 (PMC11287741; doi:10.1021/acsaenm.4c00236)
Supplement: Supplementary file 1 — em4c00236_si_001.pdf [file em4c00236_si_001.pdf]

Supporting Information

Improved Mechanical Properties of Graphene/Carbon Fibre Composites *via* Silanization

Xudan Yao†*, Jason H. Hui, Ian A. Kinloch, Mark A. Bissett*

Department of Materials, Henry Royce Institute, National Graphene Institute, University of Manchester, Oxford Road, Manchester M13 9PL, UK.

Email: x.yao@nwpu.edu.cn, mark.bissett@manchester.ac.uk

†Current Address: School of Aeronautics, Northwestern Polytechnical University, Xi'an 710072, China

S1. Thickness distribution of EEG and EEA flakes

The thickness of EEG and EEA flakes was characterized by AFM using the QI mode, with the distribution illustrated in Fig. S1, and the average values at 114.3 ± 145.3 nm and 110.5 ± 131.2 nm respectively.

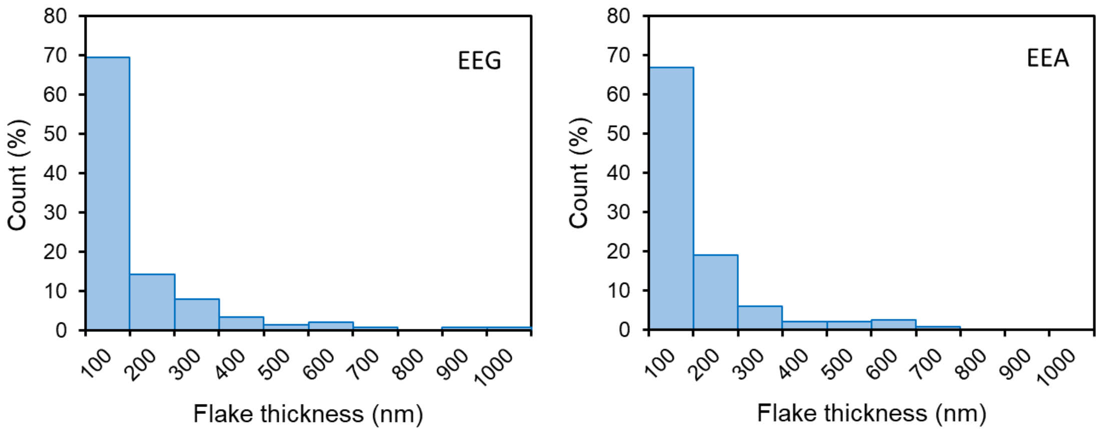

Fig. S1 Thickness distribution of EEG and EEA flakes, obtained from the AFM analysis.

S2. EDS elemental analysis of EEG and EEA flakes

Table S1 Results obtained from EDS elemental analysis of the EEG and EEA flakes.

Element	EEG (weight %)	EEA (weight %)
C	94.0	89.8
O	4.5	7.0
Si	0.15	2.6
N	0.35	0.6

S3. Raman spectra of EEG and EEA flakes

Raman mapping for both EEG and EEA have been undertaken with more than ten specimens tested for each sample. All spectra show characteristic G, 2D and D peaks, at ~1580 cm-1, ~2670 cm-1 and ~1330 cm-1, as indicated in Fig. S2. The intense G band represents the ordered graphitic structure of sp2 carbons [S1], while the D band indicates the structural defects [S2]. The intensity ratio of D peak and G peak (ID/IG) increased from 0.98 ± 0.26 (EEG) to 1.33 ± 0.21 (EEA), indicating the increased level of disorder introduced by the covalent bonding (functional groups) during the silane functionalization procedure. Meanwhile, the full-width at half-maximum of 2D peaks (FWHM (2D)) increased from 79 cm-1 (EEG) to 84 cm-1 (EEA), as a result of peaks broadening with increasing defects [S3].

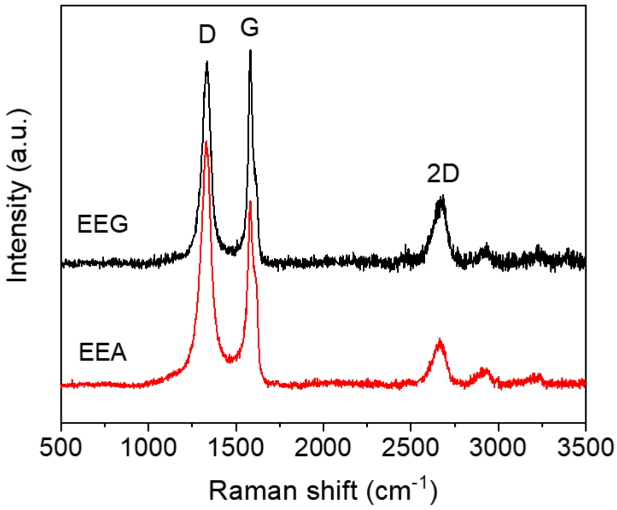

Fig. S2 Raman spectra of EEG and EEA flakes.

S4. XPS analysis of EEG and EEA flakes

Table S2 XPS elemental analysis of the EEG and EEA flakes.

	Atomic concentration (at. %)				
	C	O	Si	N	C/O
**EEG**	92.2	7.8	--	--	11.8
**EEA**	86.8	8.7	2.4	2.2	10.0

Table S3 Contributions of individual chemical bonds in the high-resolution C 1s and O 1s spectra of EEG and EEA flakes.

Sample	Name of convoluted spectrum	Characterized bonds	Binding Energy (eV)	% composition in each element
**EEG**	C 1s	C-C/C=C	284.2	86.6
		C-OH	286	1.4
		C-O-C	286.6	1.7
		π-π*	289	10.3
	O 1s	C-OH	531.7	67.1
		C-O-C	532.9	32.9
**EEA**	C 1s	C-C/C=C	284.3	72.6
		C-OH/C-O-Si	284.9	12.7
		C-N	285.3	5.7
		C-O-C	287.3	3.4
		π-π*	289.8	5.6
	O 1s	C-OH	530.6	13.2
		C-O-C/C-O-Si	532.2	76.8
		Si-O-Si	533.7	10

References

S1. Lin Y-H, Yang C-Y, Lin S-F, Lin G-R. Triturating versatile carbon materials as saturable absorptive nano powders for ultrafast pulsating of erbium-doped fiber lasers. Opt Mater Express. 2015;5(2):236.

S2. Ferrari AC, Meyer JC, Scardaci V, Casiraghi C, Lazzeri M, Mauri F, Piscanec S, Jiang D, Novoselov KS, Roth S, Geim AK. Raman spectrum of graphene and graphene layers. Phys Rev Lett. 2006;97(18):1–4.

S3. Nagyte V, Kelly DJ, Felten A, Picardi G, Shin YY, Alieva A, Worsley RE, Parvez K, Dehm S, Krupke R, Haigh SJ, Oikonomou A, Pollard AJ, Casiraghi C. Raman fingerprints of graphene produced by anodic electrochemical exfoliation. Nano Lett. 2020;20(5):3411–9.
